# Supplementary material for: Biological Synthesis, Characterization, and Therapeutic Potential of S. commune-Mediated Gold Nanoparticles
Source: Biomolecules. 2023 Dec 13;13(12):1785. doi: 10.3390/biom13121785 (PMC10741590; doi:10.3390/biom13121785)
Supplement: Supplementary file 1 [file biomolecules-13-01785-s001.zip › biomolecules-2715026-supplementary.pdf]

Supplementary File

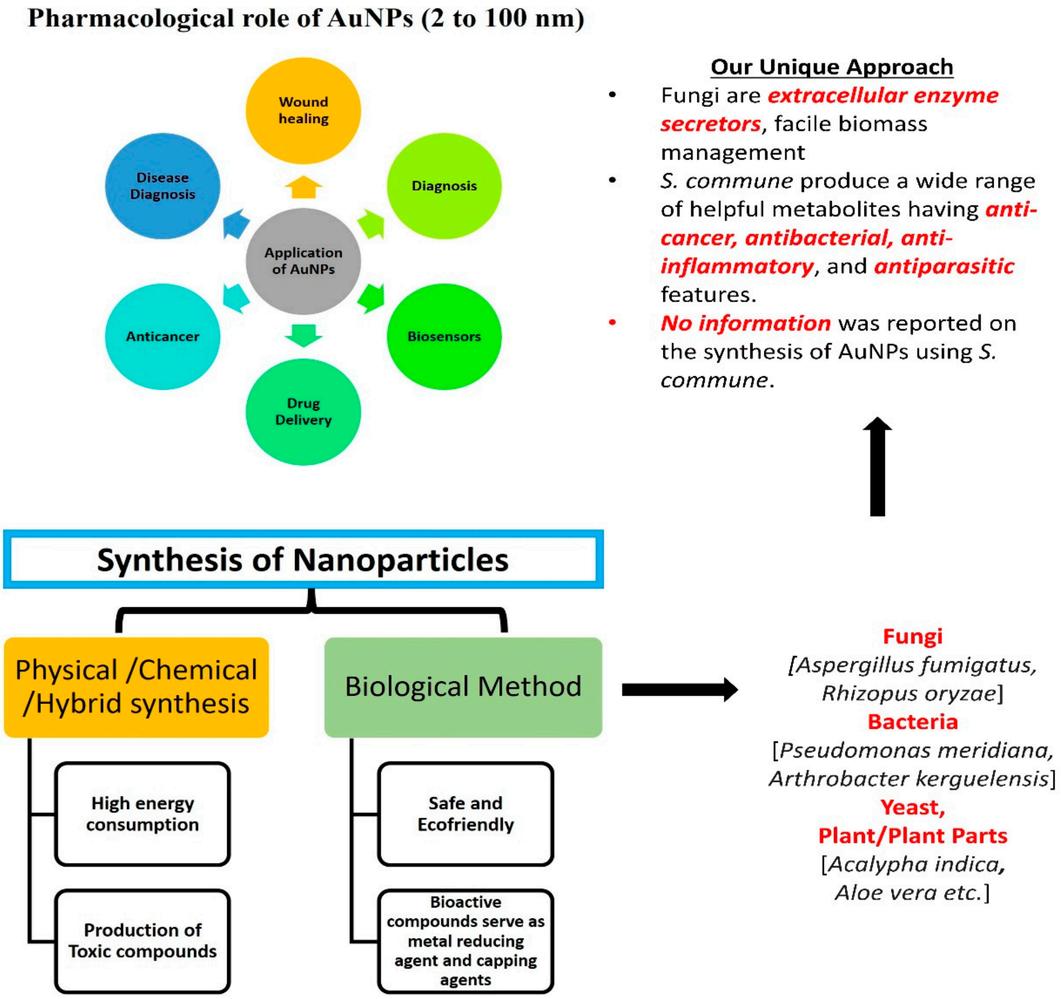

Supplementary Figure S1. Application of gold nanoparticles and its mode of synthesis

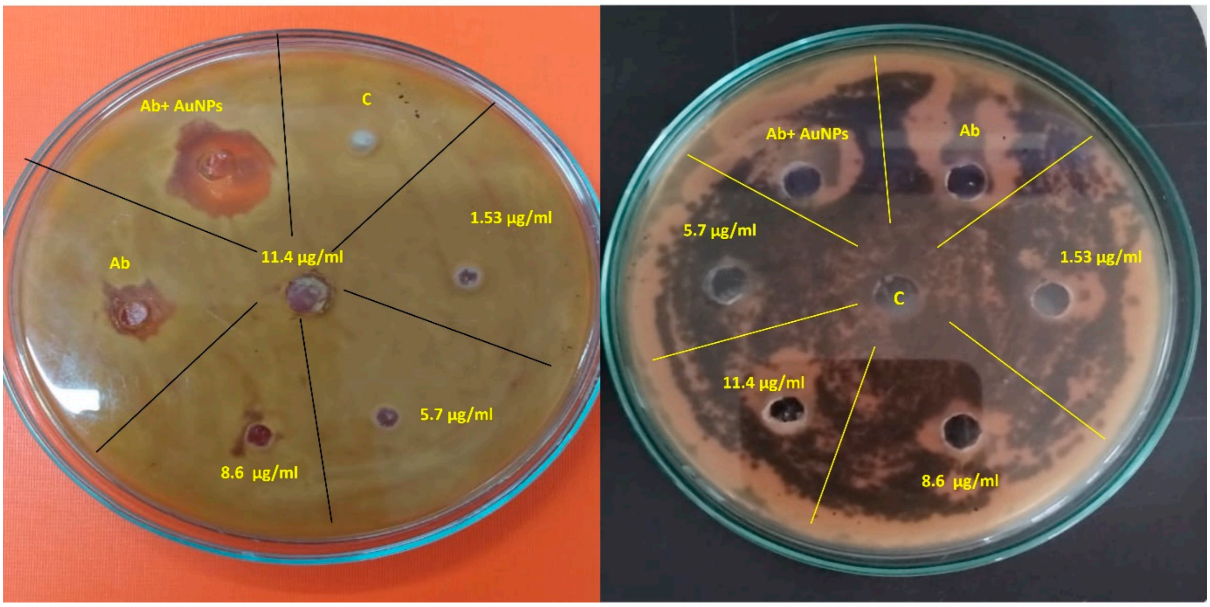

Supplementary Figure S2. Antifungal activity of gold nanoparticles (a) *Trichoderma sp*. (b) *A. flavus*
